# A Digest of Materia Medica and Pharmacy, Forming a Complete Pharmacopœia, for the Use of Physicians, Druggists and Students

**Published:** 1884-02

**Authors:** 


**A Digest of Materia Medica and Pharmacy, Forming a Complete Pharmacopœia, for the use of Physicians, Druggists and Students.** By Albert Merrell, M. D., Professor of Chemistry, Pharmacy and Toxicology in the American Medical College, St. Louis, Mo., member of the State Board of Health of Missouri. Philadelphia: P. Blakiston, Son & Co., 1012 Walnut St. 1883.

The object of the author of this volume, as stated in the preface, is to present a condensed statement of such essential facts, pertaining to each drug therein described, as will form the ground-work for their rational employment in the treatment of disease.

The work is divided into two parts, the first treating of pharmacy, and the second of materia medica. Under these two heads, the author aims to give the latest opinions of medical agents and their preparation, and much that is new in regard to their physiological action. He also endeavors to distinguish between the poisonous and the curative action of the drug, and to indicate the abnormal condition in a tissue or organ which constitutes the physical expression of the disease.

The work forms a valuable addition to our library, and will be an important guide to the practitioner in the study of the preparation of drugs and of their action and application in disease. As such we commend it to the profession.

